# COVID-19 diagnosis from chest x-rays: developing a simple, fast, and accurate neural network

**DOI:** 10.1007/s13755-021-00166-4

**Published:** 2021-10-12

**Authors:** Vasilis Nikolaou, Sebastiano Massaro, Masoud Fakhimi, Lampros Stergioulas, Wolfgang Garn

**Affiliations:** 1 Surrey Business School, University of Surrey, Alexander Fleming Rd, Guildford, GU2 7XH UK; 2 The Organizational Neuroscience Laboratory, London, WC1N 3AX UK; 3 The Hague University of Applied Sciences, Johanna Westerdijkplein 75, 2521 EN Den Haag, The Netherlands

**Keywords:** COVID-19, Chest x-rays, Artificial intelligence, Deep learning, Classification, Convolution Neural Network

## Abstract

**Purpose:**

Chest x-rays are a fast and inexpensive test that may potentially diagnose COVID-19, the disease caused by the novel coronavirus. However, chest imaging is not a first-line test for COVID-19 due to low diagnostic accuracy and confounding with other viral pneumonias. Recent research using deep learning may help overcome this issue as convolutional neural networks (CNNs) have demonstrated high accuracy of COVID-19 diagnosis at an early stage.

**Methods:**

We used the COVID-19 Radiography database [36], which contains x-ray images of COVID-19, other viral pneumonia, and normal lungs. We developed a CNN in which we added a dense layer on top of a pre-trained baseline CNN (EfficientNetB0), and we trained, validated, and tested the model on 15,153 X-ray images. We used data augmentation to avoid overfitting and address class imbalance; we used fine-tuning to improve the model’s performance. From the external test dataset, we calculated the model’s accuracy, sensitivity, specificity, positive predictive value, negative predictive value, and F1-score.

**Results:**

Our model differentiated COVID-19 from normal lungs with 95% accuracy, 90% sensitivity, and 97% specificity; it differentiated COVID-19 from other viral pneumonia and normal lungs with 93% accuracy, 94% sensitivity, and 95% specificity.

**Conclusions:**

Our parsimonious CNN shows that it is possible to differentiate COVID-19 from other viral pneumonia and normal lungs on x-ray images with high accuracy. Our method may assist clinicians with making more accurate diagnostic decisions and support chest X-rays as a valuable screening tool for the early, rapid diagnosis of COVID-19.

**Supplementary Information:**

The online version contains supplementary material available at 10.1007/s13755-021-00166-4.

## Introduction

COVID-19 is an infectious disease caused by severe acute respiratory syndrome coronavirus 2 (SARS-CoV-2) [1]. The virus was first observed in Wuhan, China, in December 2019 and then spread globally. On March 11, 2020, the World Health Organization (WHO) declared COVID-19 a pandemic [1]. The virus spreads via respiratory droplets or aerosol so that it can be transmitted into individuals’ mouth, nose, or eyes of individuals in close proximity. The most common symptoms include a high fever, continuous cough, and breathlessness [2].

COVID-19 is usually diagnosed by an RT-PCR test [3] and often is complemented by chest radiographs, including x-ray images and computed tomography (CT) scans [4]. X-ray machines are widely available worldwide and provide images quickly, so chest scans have been recommended, by some researchers [5], for screening during the pandemic. Unlike the RT-PCR test, chest scans provide information about both the status of infection (i.e., presence or absence of the disease) and disease severity. Moreover, x-ray imaging is an efficient and cost-effective procedure. It requires relatively cheap equipment and can be performed rapidly in isolated rooms with a portable chest radiograph (CXR) device, thus reducing the risk of infection inside hospitals [6, 7].

Despite these benefits, the American College of Radiology (ACR) and the Centers for Disease Control and Prevention (CDC) have not endorsed chest imaging as a first-line test for COVID-19 [8]. In the diagnosis of COVID-19, chest CT scans are highly sensitive (97%) but far less specific (25%) than RT-PCR [9]. Recent research, however, suggests that deep learning techniques may improve the specificity of x-ray imaging [10] for the diagnosis of COVID-19 (see publications in Table 1).

**Table 1:** Studies on deep learning algorithms for the detection of COVID-19 from x-rays

Study	Population size	Machine learning method	Model	Accuracy	Sensitivity	Specificity	Precision	F1-score
Chaudhary et al. (2021) [12]	14,000	CNN with transfer learning	EfficientNet-B1	95%	Three-class classification: 100% for COVID-19, 94.6% for non-COVID-19 and 95.5% for normal	N/A	Three-class classification: 94.3% for COVID-19, 96.9% for non-COVID-19, 93.5% for normal	N/A
Luz et al. (2021) [13]	13,569	CNN with transfer learning	EfficientNet-B0-B5 with 4 blocks added	From 90 to 93.9%	Three-class classification: 87%-96.8% for COVID-19	N/A	Three-class classification: 90.6–100% for COVID-19	N/A
Pharm (2021)[14]	Six datasets (n = 1124, 876, 1314, 876, 1314, 1314)	CNN with transfer learning	AlexNet, GoogLeNet, SqueezeNet	Two-class classification: > 99%, Three-class classification: > 96%,	Two-class classification: > 92% Three-class classification: > 92%	Two-class classification: > 99% Three-class classification: > 95%	Two-class classification: > 95% Three-class classification: > 94%	Two-class classification: > 94% Three-class classification: > 94%
Saiz and Barandiaran (2020) [15]	1500	CNN with transfer learning	VGG-16 SDD	94.92%	94.92%	92%	N/A	97%
Rahimzadeh & Attar (2020) [16]	11,307	Deep learning	Xception and ResNet50V2	95.5%	N/A	N/A	N/A	N/A
Panwar et al. (2020) [17]	337	Deep learning (nCOVnet)	VGG-16	88.10%	97.62%	78.57%	N/A	N/A
Li et al. (2020) [18]	2914	CNN with transfer learning	MobileNetV2	Accuracy: 96.78%	98.66%	96.46%	N/A	N/A
Sethy et al. (2020) [33]	381	CNN and SVM	ResNet-50	Not reported	95.33%	N/A	N/A	N/A
Brunese et al. (2020) [19]	6523	Deep learning (CoroNet)	VGG-16	97%	N/A	N/A	N/A	N/A
Loey and et al. (2020) [20]	306	Deep learning	GoogLeNet	100%	N/A	N/A	N/A	N/A
Ozturk et al. (2020) [21]	Not known	Deep learning	DarkNet	Binary case: 98.08%, multiclass cases: 87.02%	N/A	N/A	N/A	N/A
El Asnaoui and Chawki (2020) [22]	6087	Deep learning	Inception_ResNet_V2	92.18%	N/A	N/A	N/A	N/A
Mahmud et al. (2020) [23]	5856	Deep learning (CNN)	CovXNet	90.2%	N/A	N/A	N/A	N/A
Vaid et al. (2020) [24]	181	Deep learning (CoroNet)	VGG-19	96.3%	N/A	N/A	N/A	N/A
Ucar & Korkmaz (2020) [25]	Not known	CNN	Deep Bayes SqueezeNet	98.3%	N/A	N/A	N/A	N/A
Togaçar et al. (2020) [26]	295	Deep learning (CoroNet)	SqueezeNet and MobileNet	99.27%	N/A	N/A	N/A	N/A
Khan et al. (2020) [27]	1300	Deep learning (CoroNet)	Xception	89.6%	N/A	N/A	N/A	N/A
Yi et al. (2020) [34]	88	Deep learning (CNN)	Not known	N/A	89%	N/A	N/A	N/A
Martinez et al.(2020) [28]	240	CNN	NASNet^1^	97%	N/A	N/A	N/A	N/A
Das et al. (2020) [35]	6845	Deep learning (CNN)	Truncated inception Net	N/A	88%	100%	N/A	N/A
Waheed et al. (2020) [29]	1124	GAN (CovidGAN)	ACGAN^2^, VGG-16	95%	90%	97%	N/A	N/A
Pereira et al. (2020) [30]	1144	Deep learning (CNN)	Inception-V3	N/A	N/A	N/A	N/A	89%
Apostolopoulos et al. (2020) [31]	455	Deep learning (CoroNet)	MobileNetV2	99.18%	97.36%	99.42%	N/A	N/A
Elaziz et al. (2020) [32]	Not known (2 databases)	Deep learning (CoroNet)	MobileNet	First dataset:96.09%, Second dataset: 98.09%	N/A	N/A	N/A	N/A

^1^Neural architecture search network

^2^Auxiliary classifier generative adversarial network

Many of the studies in Table 1 use convolutional neural networks (CNNs) that take advantage of transfer learning—that is, they are built on existing CNNs (e.g., EfficientNet, VGG-16, AlexNet, GoogLeNet, SqueezeNet, ResNet) that were trained on large-scale image-classification datasets (e.g., ImageNet [11]). The size of the dataset and generic nature of the trained task (e.g., describe the general shapes of an object) make the features learned by the existing CNN useful for other computer-vision problems with other images. As shown in Table 1, most of the studies reported high accuracy (above 87%) [12–32], ten studies reported high sensitivity (above 88%) [12, 13, 16–18, 29, 30, 33–35], seven studies reported high specificity (above 78%) [14, 15, 17, 18, 28, 29, 31, 34, 35], three studies reported high F1-scores (above 94%) [14, 15, 30], and three studies reported above 94% precision [12–14]. All of the models were validated internally (i.e., trained and validated on different random splits of the same dataset; the proportions of the dataset for training versus validation vary). However, it remains unclear whether these models would perform as well on an external validation task (i.e., on a different dataset that was not involved in the model’s development).

To address the need for external validation and advance the possibility of using x-ray technology to alleviate the impact of the global pandemic, we develop a CNN using a parsimonious yet powerful pre-trained CNN as the baseline model, and we assess its diagnostic accuracy on an independent (external) dataset. We compare the model’s performance on two classification datasets: a) COVID-19 vs. normal lungs (two-class classification) and b) COVID-19 vs. other viral pneumonia vs. normal lungs (three-class classification).

## Methods

### Study design

**Fig. 1:**
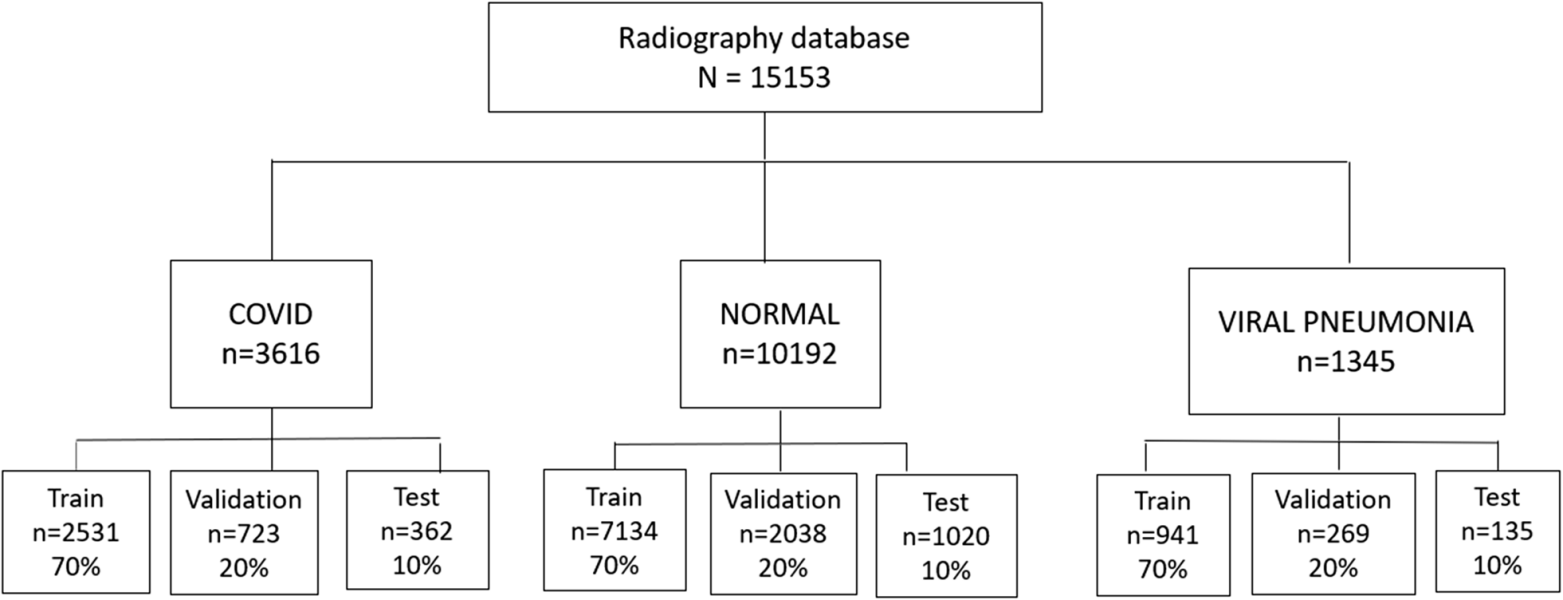
Study flow chart

We used the COVID-19 Radiography database [36], a public database that contains 15,153 images (as of 3rd May 2021) across three cohorts: COVID-19, other viral pneumonia, and normal lungs. We randomly split each of the three cohorts into train (70%), validation (20%), and test (10%) subsets (Fig. 1). Only the train and validation subsets contributed to the model’s development; we kept the test subset separate for external validation.

### Transfer learning and feature extraction

**Fig. 2:**
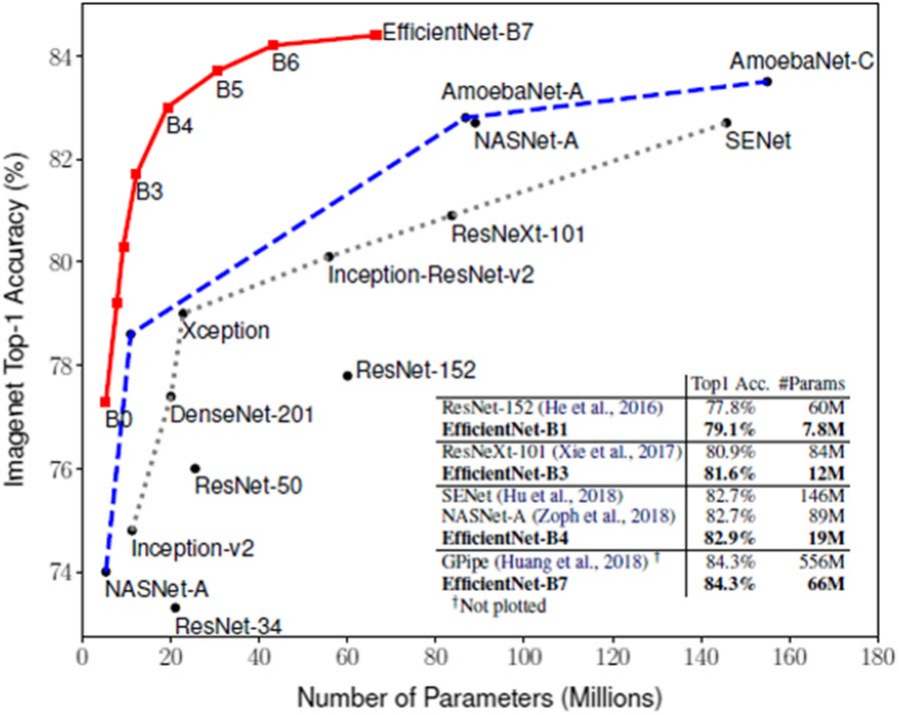
The performance of the EfficientNet models versus other CNNs on ImageNet (from Tan & Lee 2019). Source: Tan M, Le Q, Efficientnet: Rethinking model scalling for convolutional neural networks. In International Conference on Machine Learing 2019 May 24 (pp. 6105–6114). PMLR

We developed a hybrid CNN using a pre-trained ConvNet called EfficientNetB0 [37], which is the baseline model of a family of EfficientNet models (from EfficientNetB0 to EfficientNetB7). These models use compound scaling, in which the image’s dimensions (i.e., depth, width, and resolution) are scaled by a fixed amount at the same time. Models with compound scaling usually outperform other CNNs, as shown in Fig. 2: the baseline B0 model starts at a higher accuracy than some other models (e.g., ResNet-50, Inception-v2), while the latest EfficientNet model (B7) achieves the highest accuracy of all (84%). Although the EfficientNets achieve high accuracy, they require fewer parameters (5 million for B0; 66 million for B7) and less computation time than most other models. In this work, we utilize the EfficientNet-B0 as our baseline model for the following reasons: (a) it has less parameters than the rest models (B1–B7) of the EfficientNet family, (b) it is more cost-efficient for training and tuning than the more advanced EfficientNetB1-B7 model as it does not require much computational power (see Discussion section) and (c) it contributes to high accuracy in differentiating COVID-19 from non-COVID-19 viral pneumonia and healthy images (see Results section), thus satisfies the rational of our study for developing a parsimonious yet powerful convolution network.

**Fig. 3:**
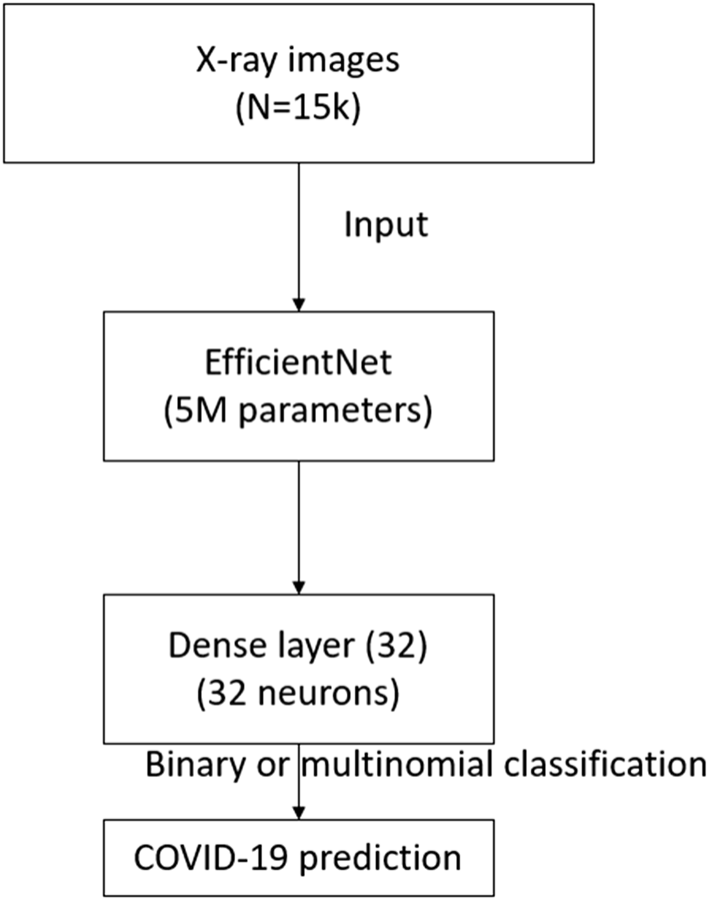
Structure of the CNN in the present study

On top of the baseline EfficientNetB0 network, we connected a fully dense layer of 32 neurons (Fig. 3). The features learned by the baseline model are run through our (two-class or three-class) classifier to extract new features from the sample.

**Fig. 4:**
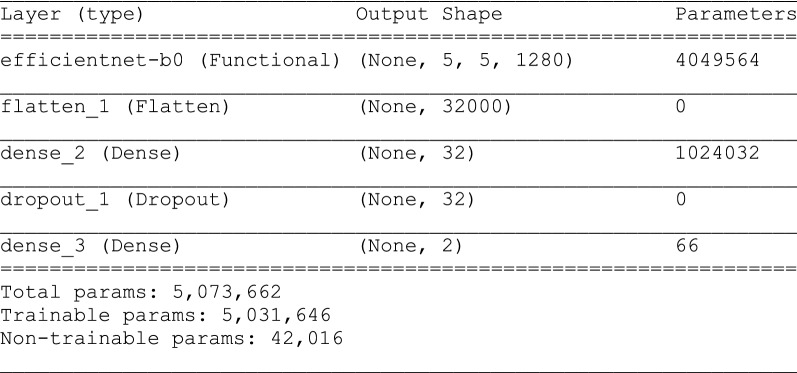
Summary of the parameters for the two-class classification

**Fig. 5:**
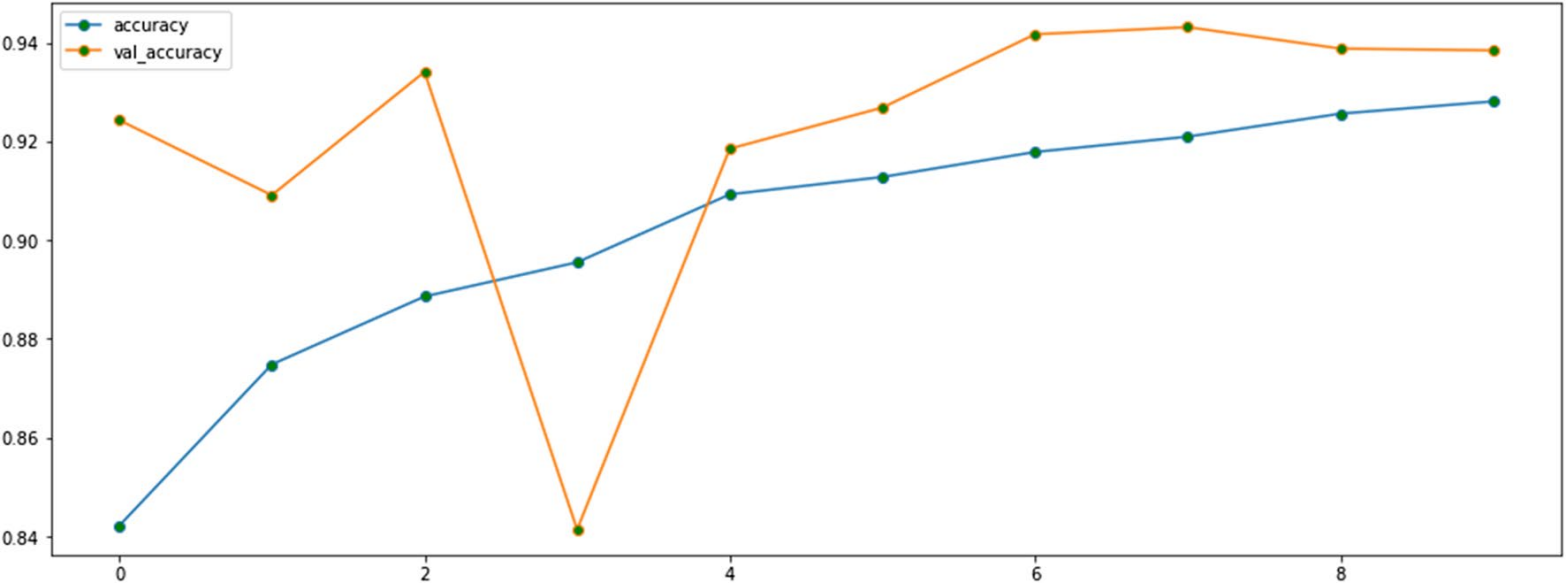
The fine-tuned model’s performance on the train and validation subsets for two-class classification

**Fig. 6:**
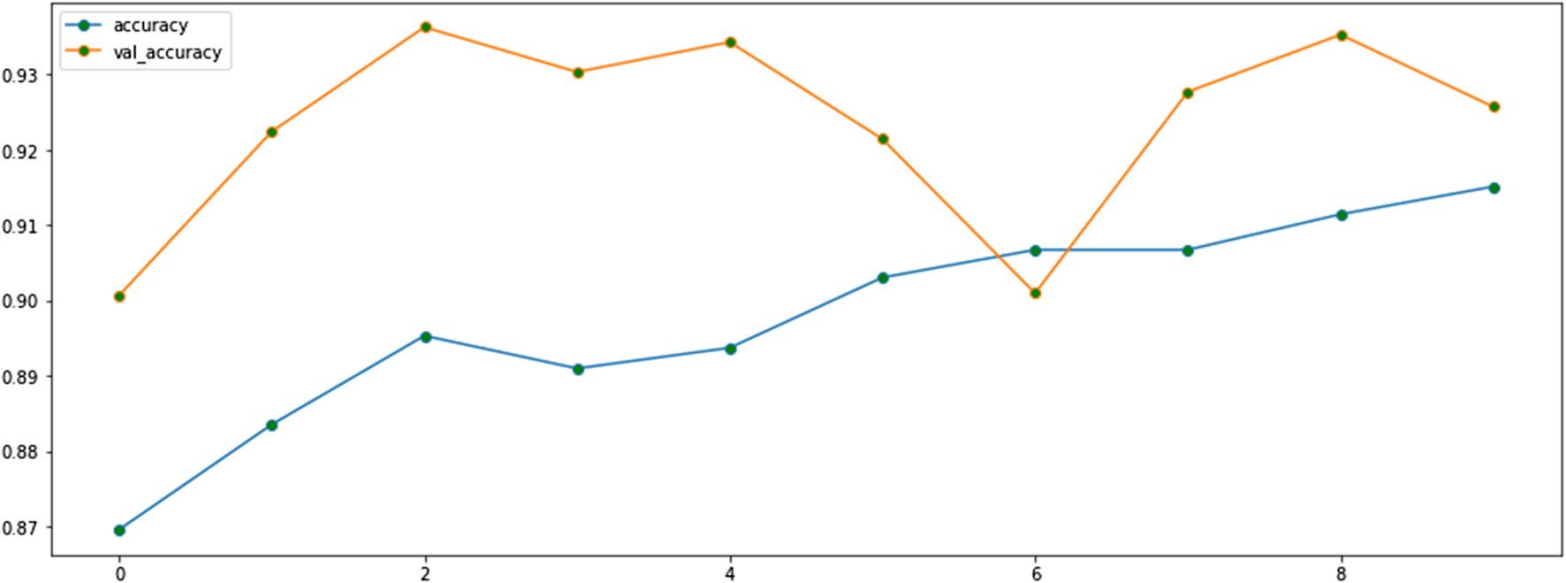
The fine-tuned model’s performance on the train and validation subsets for three-class classification

Our CNN uses about 5 million parameters (Fig. 4), which is considerably fewer than AlexNet (61 million) and GoogLeNet (7 million) [14]. Thus, our CNN was faster to train and less likely to overfit the training data, which leads to worse performance on external datasets. We also reduced the risk of overfitting by adding 20% and 50% (which outperformed the 20% drop-out rate for the three-class model; results not shown) drop-out rates to our two-class and three-class prediction models, respectively. By adding drop-out during training, a proportion of features (20% and 50% for the two- and three-class model respectively) is set to zero, whereas during validation, all features are used. This makes the model at validation more robust, leading to higher testing accuracy (Figs. 5 and 6). We applied Sigmoid and Softmax operations to model the two- and three-class classification outputs, respectively. We used an Adam optimizer with a learning rate of 0.001.

Before training the model, we “froze” the convolution base (i.e., EfficientNetB0) to preserve the representations learned during the baseline model’s original training. Subsequently, we trained only the weights in the two dense layers that we added to the convolution base.

### Data augmentation

We augmented the training data to eliminate the class size imbalance and avoid overfitting. First, we resized the images to 150 × 150 pixels to reduce the data volume, and we normalized them to the [0, 1] interval because neural networks are more efficient with small input values. Then, we augmented the training images through random transformations to increase the variety of the images. Specifically, we manipulated the parameters by (a) rotating the image by 40 degrees, (b) randomly shifted the height and width horizontally or vertically by 20% of the image’s initial size, (c) randomly clipping the image by 20%, (d) randomly zooming in by 20%, (e) randomly flipping half of the images horizontally, and (f) filling in the pixels that were created by a rotation or a height or width shift. Data augmentation is essential for avoiding overfitting in small samples because the additional, varied images prevent the CNN from being exposed to the same image twice.

### Fine-tuning

**Fig. 7:**
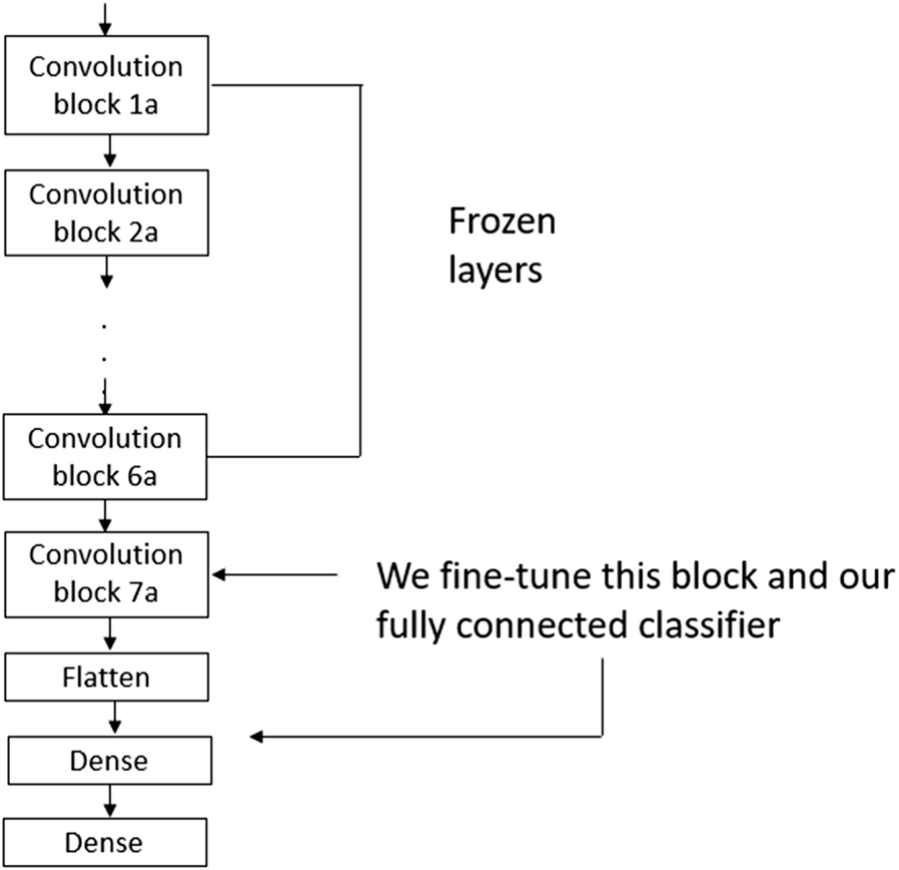
Fine-tuning the last convolution block on the EfficientNetB0 network

We fine-tuned our CNN by “unfreezing” a few of the top layers (i.e., from block 7a onwards) in the convolution base and jointly training both the “unfrozen” layers and the two layers that we added (Fig. 7). By training some of the top layers of the baseline CNN, we adjusted the presentations of the pre-trained model that were more abstract (in terms of shape and size) to make them more relevant and specific to our sample, thereby improving our model’s performance.

### Performance metrics

We assessed the model’s performance with the following metrics [14]:

(a) sensitivity (or recall): the percentage of positive cases that were predicted to be positive

$$Sensitivity= \frac{True\, Positives}{True\, Positives\, +\, False \, Negatives}$$

(b) specificity: the percentage of negative cases that were predicted to be negative

$$Specificity= \frac{True\, Negatives}{True\, negatives\, +\, False\, positives}$$

(c) positive predictive value (or precision): the percentage of positive predictions that were actually positive cases

$$Positive\, predictive\, value= \frac{True\, Positives}{True \, Positives\, +\, False \, Positives}$$

(d) negative predictive value: the percentage of negative predictions that were actually negative cases

$$Negative\, predictive \, value= \frac{True\, Negatives}{True \, Negatives\, +\, False\, Negatives}$$

(e) F1-score: a combination of recall and precision, used for model comparison

$$F1-score= \frac{2 \, X\, True\, Positives}{2 \, X \, True \, Positives\, +\, False\, Positives\, +\, False\, Negatives}$$

(f) accuracy

$$Accuracy\, =\, \frac{True\, Positives\, +\, True \, Negatives}{Total \, number \, of \, cases}$$

Moreover, we constructed 95% confidence intervals for the above metrics using bootstrapping.

## Results

We include results for the two-class classification (COVID-19 vs. normal lungs) and the three-class classification (COVID-19 vs. other viral pneumonia vs. normal lungs).

### Two-class classification

Table 2 reports the performance of the two-class classification model with feature extraction only and with fine-tuning. Figure 5 depicts the performance after fine-tuning on the train (blue line) and validation subsets (orange line). The model reached an accuracy of 93.8% on the validation subset after training for 10 epochs (i.e., runs through the train dataset).

**Table 2:** The model’s performance on two-class classification

Model with feature extraction	Predicted	
	Normal	COVID-19
Observed		
Normal	950	70
COVID-19	47	315
Accuracy (95% CI) (%)	92 (90, 94)	
Sensitivity (95% CI) (%)	87(85, 89)	
Specificity (95% CI) (%)	93 (91, 95)	
PPV (95% CI) (%)	82 (79, 84)	
NPV (95% CI) (%)	95 (94, 96)	
F1-score (95% CI) (%)	84 (82, 86)	

Model with fine-tuning	Predicted	
	Normal	COVID-19
Observed		
Normal	988	32
COVID-19	38	324
Accuracy (95% CI) (%)	95 (94, 96)	
Sensitivity (95% CI) (%)	90 (88, 92)	
Specificity (95% CI) (%)	97 (96, 98)	
PPV (95% CI) (%)	91 (89, 93)	
NPV (95% CI) (%)	96 (95, 97)	
F1-score (95% CI) (%)	90 (88, 92)	

*CI* confidence interval, *PPV* positive predictive value, *NPV* negative predictive value

As shown in Table 2, the model’s performance improved with fine-tuning, achieving a recall (i.e., sensitivity) of 90% (95% CI 88, 92), specificity of 97% (95% CI 96, 98), precision (i.e., PPV) of 91% (95% CI 89, 93), F1-score of 90% (95% CI 88, 92), and accuracy of 95% (95% CI 94, 96). The fine-tuned model misclassified only 32 normal images as COVID-19 (versus 70 misclassifications in the model with only feature extraction).

### Three-class classification

Table 3 reports the performance of the three-class classification model with feature extraction only and with fine-tuning. Figure 6 depicts the performance after fine-tuning on the train (blue line) and validation subsets (orange line). The model reached an accuracy of 92.6% after 10 epochs.

**Table 3:** The model’s performance on three-class classification

Model with feature extraction	Predicted		
	Normal	Other viral pneumonia	COVID-19
Observed			
Normal	941	0	79
Other viral pneumonia	20	108	7
COVID-19	32	0	330
Accuracy (95% CI) (%)	91 (89, 93)		
Sensitivity (95% CI) (%)	91 (89, 93)		
Specificity (95% CI) (%)	92 (90, 94)		
PPV (95% CI) (%)	79 (77, 82)		
NPV (95% CI) (%)	95 (94, 96)		
F1-score (95% CI) (%)	85 (83, 87)		

Model with fine-tuning	Predicted		
	Normal	Other viral pneumonia	COVID-19
Observed			
Normal	964	2	54
Other viral pneumonia	32	102	1
COVID-19	22	0	340
Accuracy (95% CI) (%)	93 (92, 95)		
Sensitivity (95% CI) (%)	94 (93, 96)		
Specificity (95% CI) (%)	95 (94, 96)		
PPV (95% CI) (%)	86 (84, 88)		
NPV (95% CI) (%)	95 (94, 96)		
F1-score (95% CI) (%)	90 (88, 92)		

*CI* confidence interval, *PPV* positive predictive value, *NPV* negative predictive value

As shown in Table 3, the model’s performance improved with fine-tuning, achieving an accuracy of 93% (95% CI 92, 95), recall (i.e., sensitivity) of 94% (95% CI 93, 96), precision (i.e., PPV) of 86% (95% CI 84, 88), and F1-score of 90% (95% CI 88, 92). The F1-score improved by 5% with fine-tuning relative to the model with only feature extraction. The fine-tuned model was adept at differentiating between COVID-19 and other viral pneumonia—only one other viral pneumonia image was misclassified as COVID-19, and no COVID-19 images were misclassified as other viral pneumonia. (Two normal images were misclassified as other viral pneumonia.).

## Discussion

We implemented a hybrid CNN that combines a pre-trained EfficientNetB0 network with a dense layer (32 neurons) to differentiate between x-ray images of COVID-19 and normal lungs (and other viral pneumonia, in the three-class classification). After feature extraction and fine-tuning, the model achieved 95% (95% CI 94, 96) accuracy for the two-class classification and 93% (95% CI 92, 95) accuracy for the three-class classification. This model’s performance is comparable to existing models [10, 12, 13], but it offers several other advantages.

Methodologically, to the best of our knowledge, this is the first instance in which the pre-trained EfficientNetB0 (the baseline model of the EfficientNet family, which uses compound scaling to achieve higher accuracy) with a dense layer(32) on top has been used to improve the accuracy of COVID-19 diagnosis from X-ray images. Chaudhary et al. [12] used an EfficientNet-B1 model to distinguish COVID-19 from non-COVID-19 and normal x-ray images with 95% accuracy and 100% sensitivity, while Luz et al. [13] used a range of EficientNetB0-B5 models with four dense layers on top. In the latter case, the EfficientNet-B0 with four layers on top achieved an accuracy of 90% and sensitivity of 93.5%, whereas the best performing EfficientNet-B3 with four layers on top achieved 94% accuracy and 96.8% sensitivity. In comparison, our model achieved better accuracy (93%) and slightly better sensitivity (94%) than the B0-X (where X denotes the four layers on top) with fewer parameters (5 million vs 5.3 million). It also achieved similar accuracy and sensitivity with the B1 and B3-X (6.6 and 12.3 million parameters respectively). Jiao et al. [38] also used EfficientNet as the baseline ConvNet, but the prediction was different (COVID-19 severity: critical vs. non-critical), and the model was considerably more complex: it connected a convolutional layer (256 neurons) and a dense layer (32 neurons) on top of the baseline. Despite its complexity, the model did not perform as well on an external dataset: accuracy of 75% (95% CI 74, 77), sensitivity of 66% (95% CI 64, 68), and specificity of 70% (95% CI 69, 71). Although the present model addresses a different problem, we are confident that it performs better on the two-class classification problem than the model built by Jiao et al. Given that a model trained on more parameters is more prone to overfitting, we believe that our relatively small number of parameters and data augmentation strategy contributed to our model’s superior performance.

Another advantage of this study lies in its design. Previous works have used a variety of splits to create subsets of data for two purposes: training and validation. For instance, Pham [14] used several random splits (80% vs. 20%; 50% vs. 50%; 90% vs. 10%) of an older version of the COVID-19 Radiography database used here. Rahimzadeh et al. [16] used eight subsets of their dataset for training and another subset for testing; Panwar et al. [17] used 70% for training and 30% for validation. To the best of our knowledge, the present research is the first to cluster the dataset into three independent subsets: train (70%), validation (20%), and test (10%). Thus, we reduced overfitting by testing the model’s performance on a subset of data that did not contribute to the model’s development.

We also mitigated overfitting by augmenting the training images (i.e., randomly transforming existing images to generate new ones). Data augmentation also addressed the class imbalance (as there were unequal numbers of COVID-19, other viral pneumonia, and normal images). We fine-tuned the model by training some of the top layers of our baseline CNN to improve their specificity to the current problem.

Finally, all of the reviewed studies except for one [14] reported only point estimates for their performance metrics (accuracy, sensitivity, specificity, positive predictive value, negative predictive value, and F1-score). By including the 95% confidence intervals, we capture the uncertainty of our estimates and enable a more comprehensive appraisal of the model.

The main limitation of our study is the lack of patient data. We appreciate that the model built by Jiao et al. [38] included patient clinical data (e.g., age, sex, oxygen saturation, biomarkers, comorbidities), which slightly improved the accuracy of the image-based CNN. We recognize that this is an important avenue for future research. We further note that the training and tuning of our “light” CNN took about two hours on a conventional Macintosh computer with 16G RAM and one Terabyte of a hard disk. More computational power is instead needed to train more advanced versions of the EfficientNet (from B1 through B7), possibly increasing the barrier of entry for non-specialists users (i.e., clinicians).

## Conclusions

This study uses a “light” CNN to discriminate COVID-19 from other viral pneumonia and healthy lungs in chest X-ray images. To the best of our knowledge, our model is at present the most parsimonious CNN used to address the demanding issue of COVID-19 diagnosis via chest x-ray. The model successfully overcame the issue of low specificity that has prevented the ACR and CDC from endorsing chest imaging to diagnose COVID-19 [8]. Specifically, the fine-tuned model differentiated COVID-19 from normal lungs with a positive predictive value of 91% and specificity of 97% (95% CI 96, 98); it differentiated COVID-19 from other viral pneumonia and normal lungs with a positive predictive value of 86% and specificity of 95% (95% CI 94, 96). Both classifications had a negative predictive value of 95% (95% CI 94, 96), meaning that a negative COVID-19 classification would indicate a 95% chance of not having COVID-19. Moreover, as shown in Table 3, just one image of other viral pneumonia was misclassified as COVID-19, proving a remarkable discriminatory ability given the overlap in the presentation of COVID-19 and other viral cases of pneumonia.

The insights presented in this study may help clinicians (namely, radiologists) accurately diagnose COVID-19 at an early stage by enabling the use of x-ray as a first-to-test tool that can complement RT-PCR analyses. Moving forward, the validation of our CNN on other databases would increase our confidence in the use of neural networks to aid the early diagnosis of both COVID-19 and other life-threatening diseases that traditionally have been difficult to diagnose via imaging.

## Supplementary Information

Below is the link to the electronic supplementary material.

Supplementary file1 (DOCX 40 kb)

## Data Availability

The publicly available COVID-19 Radiography Database [36] was used.

## Code availability

The python notebook for the two-class classification model is available here: http://localhost:8888/notebooks/OneDrive%20-%20University%20of%20Surrey/Kaggla_data/Two-class_classification.ipynb. The python notebook for the three-class classification model is available here: http://localhost:8888/notebooks/OneDrive%20-%20University%20of%20Surrey/Kaggla_data/Three-class_classification.ipynb.
