# Supplementary figures and images for: COVID-19 diagnosis from chest x-rays: developing a simple, fast, and accurate neural network
Source: Health Inf Sci Syst. 2021 Oct 12;9(1):36. doi: 10.1007/s13755-021-00166-4 (PMC8509906; doi:10.1007/s13755-021-00166-4)

Figure 7.1 Training and validation loss for three-class classification model

*
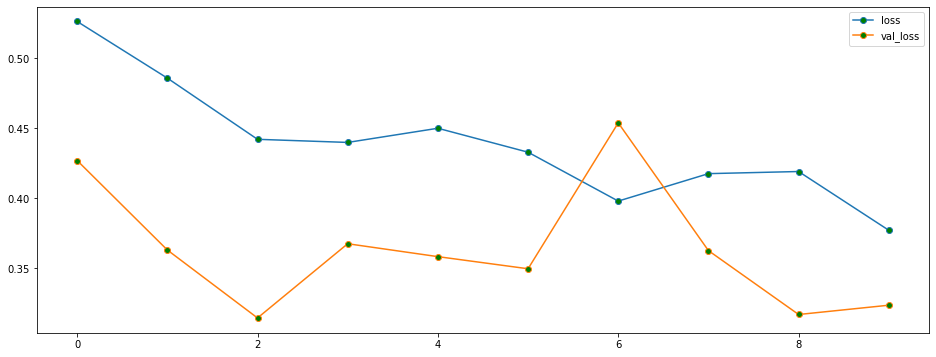
*

Supplement: Supplementary file 1 — Supplementary file1 (DOCX 40 kb) [file 13755_2021_166_MOESM1_ESM.docx]
